# Radiological Influencing Factors in the Diagnosis of Painful Habitual Instability of the Thumb Basal Joint as a Precursor of Carpometacarpal Arthritis of the Thumb—A Retrospective Study

**DOI:** 10.3390/jpm13050704

**Published:** 2023-04-22

**Authors:** Raimund Winter, Sophie Hasiba-Pappas, Lars-P. Kamolz, Sebastian Tschauner, Oskar Bamer, Alexandru Cristian Tuca, Hanna Luze, Sebastian P. Nischwitz, Birgit Michelitsch, Herwig Friedl, David Benjamin Lumenta, Werner Girsch

**Affiliations:** 1 Research Unit for Tissue Regeneration, Repair and Reconstruction, Division of Plastic, Aesthetic and Reconstructive Surgery, Department of Surgery, Medical University of Graz, Auenbruggerplatz 5, A-8036 Graz, Austria; 2 COREMED—Cooperative Centre for Regenerative Medicine, Joanneum Research GmbH, Neue Stiftingtalstr. 2, A-8010 Graz, Austria; 3 Division of Pediatric Radiology, Department of Radiology, Medical University of Graz, A-8036 Graz, Austria; 4 Department of Orthopedics and Trauma Surgery, Division of Trauma Surgery, Medical University Vienna, A-1090 Vienna, Austria; 5 Institute of Statistics, Graz University of Technology, Kopernikusgasse 24, A-8010 Graz, Austria

**Keywords:** thumb basal joint arthritis, joint laxity, CMC-1 arthritis, plastic surgery, radiographic imaging, hand surgery

## Abstract

Background: Painful habitual instability of the thumb basal joint (PHIT) is a rarely diagnosed condition that can severely impair hand function. Furthermore, it can increase the risk of developing carpometacarpal arthritis of the thumb (CMAOT). Clinical examination and radiographic imaging provide the foundation for a correct diagnosis, but early detection is still challenging. We investigated two objective, radiographically obtainable parameters as potential risk factors for PHIT. Methods: Clinical data and radiographic images of 33 patients suffering from PHIT were collected and compared to those of 35 people serving as the control group. The two main objectives, the slope angle and the bony offset of the thumb joint, were gathered from the X-rays and statistically analyzed. Results: The analysis showed no differences between the study and the control group concerning the slope angle. Gender and the bony offset, on the other hand, had a significant influence. Female sex and higher offset values were associated with an increased risk of PHIT. Conclusions: The results of this study prove a connection between a high bony offset and PHIT. We believe this information can be valuable in early detection and will allow more efficient treatment of this condition in the future.

## 1. Background

The thumb is the most important finger of the hand. Any restrictions concerning the thumb joint lead to a drastic reduction of the entire hand’s function and should therefore always be taken seriously [1].

One of the most common conditions leading to thumb impairment is “rhizarthrosis”, also known as “carpometacarpal arthritis of the thumb” (CMAOT). Approximately one in four women and one in twelve men show arthritic changes in the thumb saddle joint in radiographic images [2]. 

A variety of factors can contribute to the development of CMAOT [3,4]. One of them is a lack of joint stability, which is often the result of insufficient support provided by ligaments [5]. Sixteen ligaments are involved in the stabilization and function of this crucial joint [6,7]. It has been reported that these ligaments contain a number of hormone receptors, such as estrogen and relaxin receptors. These two hormones are thought to be involved in increasing ligament—and consequently joint capsule—laxity [8]. 

Instability of the joint as a result of lax bands may cause CMAOT, but it can also mimic this condition by showing similar symptoms without the typical arthritic/arthrotic changes [9]. This is the so-called painful habitual instability of the thumb basal joint (PHIT). In the case of PHIT, early treatment should be considered to prevent joint destruction in the future [10].

The diagnosis of ligamentous laxity is primarily established through clinical examination or using relatively complicated bilateral dorso–palmar/anterior–posterior stress test radiographs of the thumb, which are rarely performed in the outpatient setting of routine daily clinical practice. While demonstrating joint laxity, trapeziometacarpal joint stress images are unable to distinguish between painless constitutional laxity and painful pathologic laxity [11]. Thus, there is no objective tool to detect PHIT at an early stage.

Two objective radiological signs, the bony offset and the slope angle, have gained attention in the diagnostic evaluation process of patients suffering from PHIT [3,10,12]. The slope angle was first described by Kapandji et al. [3] in 2002. It is the angle formed between the axis through the os metacarpale II and the axis through the os trapezium. Physiologically, this angle should be between 123° and 135°. Values over 135° are interpreted as trapezium dysplasia [3]. The second parameter, the bony offset of the os metacarpale I, is measured in relation to the trapezium. Both can easily be determined by examining standard radiographs in dorso--palmar and zither player positions.

The aim of this retrospective study was to investigate whether bony offset and slope angle can be associated with PHIT, and possibly identify these parameters as risk factors for thumb joint instability. The results of this research should help in diagnosing PHIT early on and thus contribute to the early detection of this rare disease and possible allow prevention of manifest carpometacarpal arthritis of the thumb.

## 2. Materials and Methods

The study was performed after obtaining two ethical approvals (Medical University Vienna and Medical University Graz; institutional review board EK18-083-VK and EK35-039 ex 22/23) and in accordance with the rules set by the Declaration of Helsinki.

The purpose of this retrospective data analysis was to determine if the slope angle and bony offset of the first metacarpal bone are risk factors for developing instability in the thumb saddle joint—thereby contributing to arthralgia, loss of strength and CMAOT. We investigated if a relationship between PHIT and slope angle or PHIT and bony offset can be proven. Clinical examination and radiographic imaging in two planes, dorso–palmar image and zither player projection, had been performed in all patients.

### 2.1. Inclusion and Exclusion Criteria of the Study Population

Patients between the ages of 15 and 51 who suffered from painful habitual instability and underwent surgery at the Orthopedic Hospital Speising, Vienna, between 2010 and 2017 were included in the study. Patients with radiologically proven open growth plate, preexisting CMAOT, rheumatoid disease, congenital deformities of the thumb or a medical history of traumatic injuries to the thumb joint were excluded.

### 2.2. Inclusion and Exclusion Criteria of the Comparison Group

Patients for the control group were recruited at the Orthopedic Hospital Speising in Vienna, as well as at the Clinical Department of Plastic, Aesthetic and Reconstructive Surgery Graz between 2010 and 2022. The comparative collective consisted of patients that were examined at the out-patient clinic at one of the study centers and presented without signs of painful thumb saddle joint instability or CMAOT. Pre-existing hand X-rays in dorso–palmar and zither player position must have been available from these patients to be included in this study. This comparative collective is considered a sample of the healthy population.

### 2.3. Measurements

All patients were clinically evaluated for discomfort in the thumb saddle joint. Instability was determined by provocation of joint subluxation in the clinical exam.

Radiographic imaging of the hand in the dorso–palmar and the zither player positions was obtained from patients from both groups.

**Figure 1:**
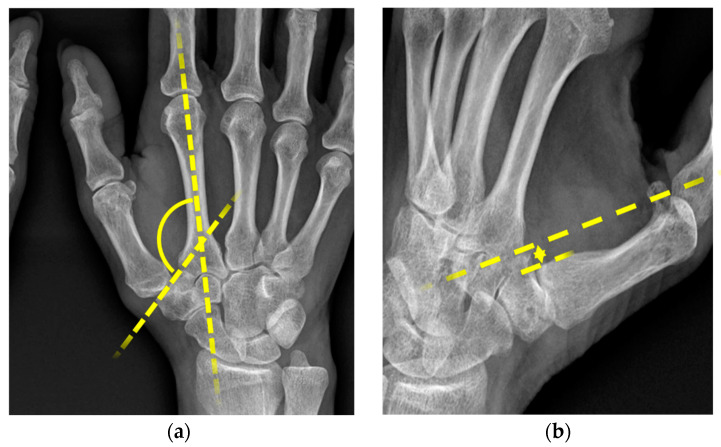
(**a**) Slope angle ^1^, (**b**) bony offset ^2^. ^1^ radiological measurement of slope angle in DP images, formed by the axis through the os metacarpale II and trapezium; ^2^ radiological measurements of width offset in zither player position (width between the lines along the ulnar edge of the trapezium and ulnar edge of the os metacarpale I).

The absolute values for the slope angle defined by Kapandji 2002 [13] and for the bony offset of the os metacarpale I were determined. All measurements were performed by an experienced radiologist. The parameters collected for this study are displayed in Figure 1a,b.

### 2.4. Statistical Analysis

The statistical analysis was performed using R-4.2.2, a free software environment for statistical computing and graphics [14]. Wilcoxon rank sum tests were applied to check for differences in the medians of two groups. Relevant predictors (influential variables or multilevel factors) in the logistic models were found by applying a backward variable elimination procedure. Linear and multilevel predictors were finally assessed through likelihood ratio tests based on the respective deviance changes. All tests related to two-sided hypotheses and *p*-values below 0.05 were considered statistically significant.

## 3. Results

Our study group consisted of 33 patients and 56 thumb saddle joints. The patient collective was predominantly female, as 30 (90.9%) recruits were women and only 3 (9.1%) were men, as shown in Table 1. A total of 35 patients and 56 thumb saddle joints were included in the comparison group. Twenty-two (62.9%) were male and thirteen (37.1%) were female. The mean age was quite similar in both groups with an average age of 35.6 (s.e. 11.5) years in the study collective and 34.7 (s.e. 10.7) years in the comparison group.

The mean slope angle was 139.0 (s.e. 9.4) in the study group and 139.9 (s.e. 9.6) in the comparison group. 

The mean offset of the os metacarpale was 2.03 (s.e. 2.31) in the study collective and 0.79 (s.e. 1.35) in the comparison collective. 

**Figure 2:**
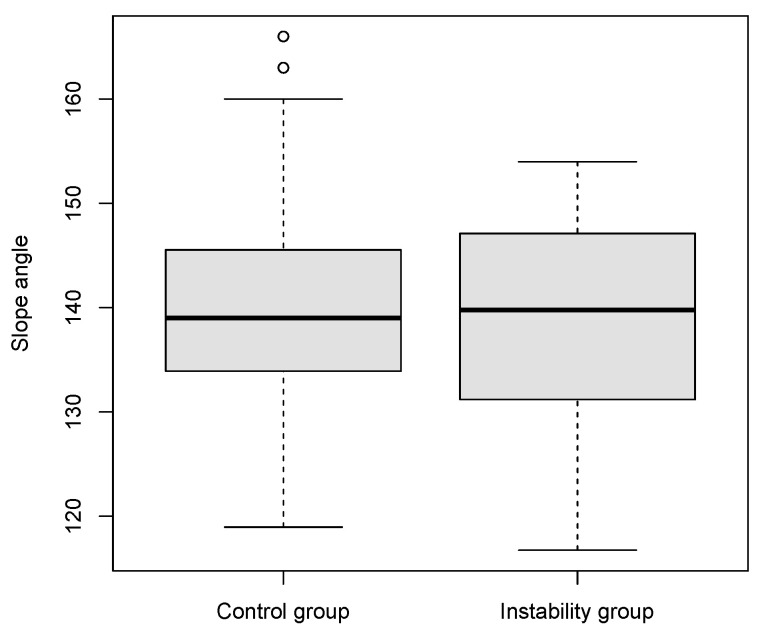
Slope angle values for the study group versus control group presented as boxplots.

No significant difference could be proven between the median slope angles in the two collectives (*p* = 0.94) (Figure 2).

However, there were noticeable differences between the study group and the comparative collective concerning the median width offsets (*p* = 0.047). This was further analyzed using a logistic regression model, which showed that female gender (*p* < 0.001) and a high bony offset (*p* = 0.014) were correlated with a higher risk for joint instability.

To get a more detailed insight into the connection between width offset and PHIT, we further calculated the risk profile for patients presenting with a bony offset of 0, 2, 4 and 6 mm. 

**Figure 3:**
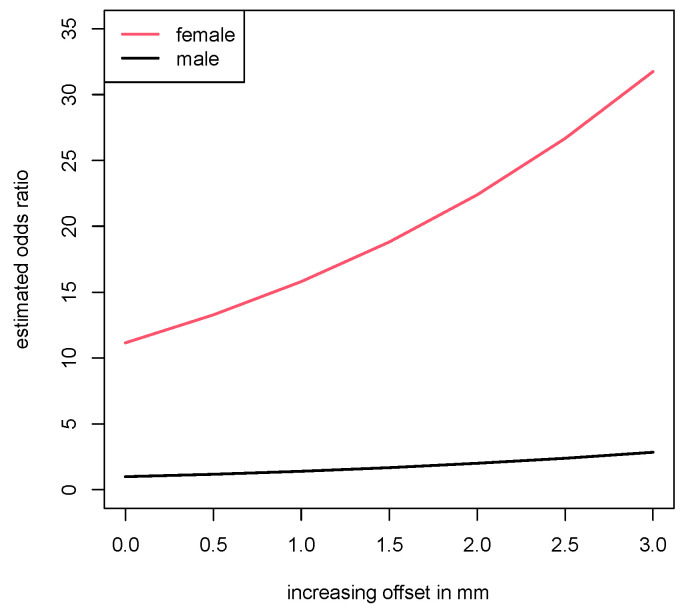
Odds ratio for PHIT with increasing offset values.

The results showed that women had an 11.1-fold higher risk of developing PHIT, compared to men. As for the offset, the analysis revealed that the odds ratio of a person with unit offset, as compared to a person with zero offset, was 1.417. With each additional mm in width of the offset, this value increased quadratically. Therefore, the odds at offset values of 4 mm were about twice as high as the odds at 2 mm. These findings are shown in Figure 3.

**Figure 4:**
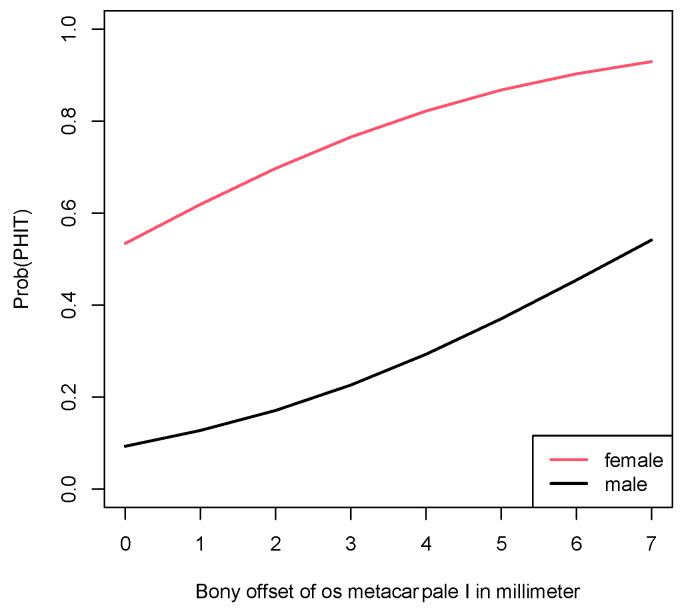
Probability for PHIT according to gender and bony offset.

PHIT probabilities for men and woman corresponding to various bony offset values are provided in Table 2. These estimated probabilities stem from the logistic regression model and are also displayed in Figure 4.

**Figure 5:**
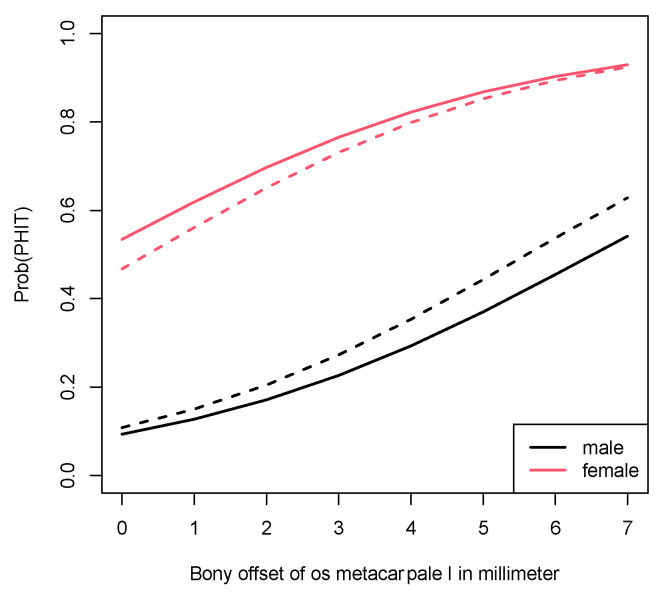
Model comparing the probability for PHIT for (**a**). gender distribution in this study (continuous lines) to (**b**). equal female-to-male ratio (dashed lines).

The distribution of female and male patients was unequal in the study group and the comparative collective. However, this factor did not diminish the results concerning the probability for PHIT, as demonstrated in Figure 5. It shows the probability for PHIT in a model in which the weighting of the genders was adjusted. In this model displaying the probability in the case of an equal female-to-male ratio, the estimated probability of developing PHIT was still considerably higher for women.

## 4. Discussion

Habitual instability of the thumb saddle joint is considered a risk factor for the development of CMAOT. It can cause the same symptoms as CMC-1 joint arthrosis even without typical radiographic signs. Due to constant pain, restriction of movement and ultimately significantly decreased hand function, patients are severely limited in their everyday life.

A high number of patients develop these symptoms at a young age. They are associated with genetic abnormalities that lead to general hyperlaxity of the connective tissue and hypermobility of the joints [5,10,15].

Similar to manifest CMC-1 osteoarthritis, therapeutic approaches range from various conservative treatment options to surgical procedures. Non-operative treatment depends on the severity of the symptoms. Mild to moderate cases may be treatable with analgesics, splinting or physical therapy. If patients do not respond to either of these methods, corticosteroid injections can be administered to provide relief for about 2 to 3 months [11]. These approaches can reduce pain or inflammatory processes temporarily. However, they can never fully restore joint stability due to the irreversible laxity of the ligaments [16]. Ligament reconstruction can be achieved surgically by performing ligamentoplasty. A variety of techniques have been described in the literature, none of which have shown clear superiority over other methods [15,17]. 

One of the first ligamentoplasty attempts was reported in 1943. For this surgery, a transosseus tendon transplant was used to stabilize the CMC-1 joint [18]. Although the pain and range of motion were improved shortly after surgery, the positive effects could not be sustained long term [18,19]. In the following years, Eggers reported on his approach for joint stabilization by splitting the musculus extensor carpi radialis longus and reattaching part of it to the ulnar side of the base of the CMC-1 joint [20]. However, this technique is prone to dislocation due to the traction on the tendon. Kestler et al. [21], on the other hand, applied intra-articular stabilization by transosseus ligamentoplasty via the extensor pollicis brevis tendon. This technique actually showed similarities to the first approach taken by Slocum et al. [18]. Michele published his experience with transarticular tendon transplants using bur holes close to the joint [21]. The m. abductor pollicis longus was once again used for joint stabilization according to a case report by Cho in the 1970s [20,22,23]. He reportedly relocated the tendon to the volar part of the trapezium bone. The patient described in this case report supposedly showed a full range of motion and was pain-free 18 months after the procedure had taken place. 

Some of the most established experts in the field of carpometacarpal surgery, Eaton and Littler et al. [24], developed a technique for joint reconstruction in 1973, which has since been refined and modified and is, to this day, one of the most widely applied techniques [24,25,26]. The chosen access for this operation is the palmar side of the hands. After drilling bur holes through the base of the os metacarpale I, part of the m. flexor carpi radialis is pulled through the bur hole, wrapped around the abductor pollicis longus and flexor capri radialis tendons and sutured onto the base of the abductor pollicis longus tendon. The author developed this technique based on the assumption that the anterior oblique ligament plays a crucial role in sustaining joint stability [23]. This procedure, though quite complex, yielded promising results in a study in 1973, as well as in more recent studies, and has maintained its status as the preferred treatment for chronic thumb joint instability until today [20,27,28,29].

At the end of the 1980s, Brunelli used the abductor pollicis longus tendon to stabilize the thumb joint by suturing it onto the extensor carpi radialis tendon after placing bur holes in the os metacarpale I and II [28]. This technique was modified by Botelheiro et al. [29] in 2001, due to the risk of nerve damage to the branches of the radialis nerve. He used the tendon of the palmaris longus muscle instead of the abductor pollicis longus, as described before, and sutured it onto the extensor carpi radialis tendon.

In 2006, Ozer et al. [30] published his ligamentoplasty approach, which entailed transosseus access through the os metacarpale I, II and the os trapezium as well as part of the extensor carpi radialis brevis tendon, which is attached to the abductor pollicis longus tendon [30]. 

While the etiology of the CMC-1 injuries varied among the aforementioned studies, Langer et al. [20] published research on a predominantly female study collective (71% of the patients were women) with idiopathic thumb joint instability in 2015. Only a few of the participants, most of whom were male, presented with CMC-1 instability as a result of traumatic injuries. The authors operated on 24 patients over the course of 12 years. For this surgical method, the pedicled abductor pollucis longus tendon is threaded through a V-shaped bur hole in the trapezium and finally attached onto its own base. The patients were highly satisfied with the procedure and presented with subjectively improved strength in the affected hand. Half of the patients received a follow-up examination two years post-operatively, at which none of them showed any signs of carpometacarpal arthritis of the basal thumb joint [20]. 

The position of the os trapezium, which is the center of our study, was first investigated by Kapandji et al. [13] in 2002. He attempted to stabilize the thumb basal joint by performing an open wedge osteotomy on the trapezium, thereby correcting dysplasia of the carpal bone and achieving a physiological slope angle.

The anatomy of the trapezium, in terms of dys-/hypoplasia was also discussed by Stauffer et al. [10] in 2019. The authors reported on CMC-1 stabilization with the use of an abductor pollicis longus tendon strip in 12 patients with chronic habitual instability of the basal thumb joint. Unlike reports from most authors, the surgeons abstained from placing bur holes and instead created deep transverse tunnels through the CMC joint via sharp incisions. After careful preparation and dissection, the abductor pollicis longus tendon is mobilized and followed to the insertion point. Half of the tendon strip is pulled through the tunnels, forming a loop resembling the shape of an “8”, and sutures are placed throughout to secure the knots and guarantee stability. Finally, the authors performed a passive shift test to confirm that the joint remains stable under traction. The motive for foregoing bur holes was to avoid unnecessary trauma to the patients’ growth plate, since instability can occur even at a young age and patients might still have open growth plates.

The results from this study showed promising outcomes in mid-term follow-ups in terms of pain reduction and DASH (disability of the arm, shoulder and hand score) improvement. Moreover, post-operative morbidity was very low, and only one case of instability recurrence was reported in the mid-term follow-up examination [10]. Radiographic evaluation of the patient collective showed that participants who presented with trapezium hypoplasia—defined in this study as an increased slope or tilt of the trapezium or abnormal bone width—were more likely to show severe radiologic shift during stress testing than participants with physiological trapezium positions. In total, 27 patients included in our study underwent prophylactic surgery for PHIT symptoms according to the technique described by Stauffer et al. [10]. The ligamentoplasty technique used in this study was first applied by Dr. Girsch and Dr. Weigel at the Orthopedic Hospital in Speising, Vienna.

Overall, ligamentoplasty yields good results and may prevent joint degeneration [20]. In spite of the encouraging results, there is no guarantee that performing these procedures will improve the symptoms, nor that the patients will not develop CMAOT in the future. That is why we believe it is not only important to educate patients about their condition, but also about the possible outcomes of the different treatment options and the fact that they may not be curative. Still, improving patient satisfaction should always be strived for even if the symptoms cannot be completely resolved.

One step towards a higher satisfaction rate in affected patients is the early detection of PHIT. The diagnosis of habitual instability is primarily made clinically, which is why performing the clinical examination of the hand thoroughly is crucial. The typical clinical sign is successful joint subluxation after provocation. A healthy, stable CMC-1 joint would allow zero to very little movement of the base of the os metacarpale I. In the case of instability, the joint space is easily widened when manual traction is applied [20]. 

Radiographic images, such as standard bilateral X-ray radiographs in dorso–palmar and zither positions, are part of the diagnostic process as well. Dorso–palmar stress radiographs, first introduced by Eaton and Littler [24], are better suited in the diagnosis of PHIT than standard X-rays. For these images, the radial borders of the distal phalanges are pressed together, thereby exposing subluxation in the saddle joint. This way, the degree of instability, in relation to the contralateral joint, can be assessed [15]. However, we find that these images can be quite unreliable and sometimes make it difficult to verify the findings objectively. This is especially challenging in the case of painful yet not radiographically severe instability.

Cinematography presents a more distinctive and reliable diagnostic option compared to other X-ray images. It allows continuous radiographic evaluation and documentation of motion sequences. Conditions that require dynamic radiographic analyzation of movement patterns, such as instability, joint dislocation and limited joint mobility, are indications for cinematography [31]. Unfortunately, this dynamic examination method is not only expensive and time-consuming, but also requires a lot of personnel.

A solid diagnosis should be confirmed prior to any surgical treatment since joint instability can be hard to verify postoperatively and may not be as detectable. For this reason, a thorough preoperative workup with confirmation of joint laxity should be conducted pre-operatively without fail. This includes the use of the aforementioned imaging diagnostics in addition to the clinical examination. 

A recurring issue in outpatient care, in our experience, is that even though various advanced diagnostic methods are discussed in literature and applied in the context of clinical studies [10,20,31], they are rarely implemented in daily clinical settings. This may be attributed to factors like complexity or cost of the procedure, or the lack of time and personnel to perform them. Standard radiographs, however, are part of any routine examination for patients presenting with hand pain in an outpatient setting. As we have established, both the slope angle and the bony offset can be obtained from said radiographs. 

Our research indicates that the offset of the os metacarpale I is a contributing factor to PHIT. Implementing evaluation of this radiological sign in routine examination of hand X-rays—essentially “screening” patients for pathological offset values—could be an asset in early detection of thumb joint laxity and therefore improve patientcare. Furthermore, gender appears to play a significant role in PHIT development, as female sex correlated with a higher risk for instability. Generally speaking, ligamentious laxity and subsequent joint instability tend to occur more often in female patients. This might be attributed to the presence of hormone receptors for estrogen and relaxin in the ligaments, as relaxin is believed to weaken ligament stability [8]. However, anatomical differences between the two sexes could also impact the gender distribution of PHIT. For one, the surface of the trapezium is often flatter in women compared to men, resulting in diminished joint congruence [32,33]. Furthermore, it has been observed that women tend to have a thinner cartilage layer than men. This presumably leads to increased contact stress in the joint, which might also explain the high prevalence of CMC-1 arthritis in female patients [32]. Carpometacarpal arthritis of the basal thumb joint, in our experience, is predominantly found among post-menopausal women. However, the findings of the present research indicate that unlike in the case of CMC-1 arthritis, a higher age does not constitute a risk factor in PHIT development as the patients included in this study were quite young—with an average age of 35.6 in the study population. This was also observed by Stauffer et al. [10], who operated on 12 patients suffering from PHIT. The average age in this study was only 23.2.

The aim of the present study was to investigate a possible association between two easily obtainable parameters, the slope angle and bony offset of the CMC-1 joint, and PHIT. Our research showed that high offset values corresponded with increased likelihood of basal thumb joint instability. According to our analysis, the probability of developing painful thumb joint instability increased from 0.534 (offset 0 mm) to 0.822 with offset values of 4 mm for the female patients included in this study. For the male population, the probability for PHIT was 0.093 at a 0 mm offset and 0.293 at a 4 mm offset. This confirms our hypothesis that the bony offset serves as a precursor for PHIT and potential subsequent CMAOT. It is an objective and reliable factor that can be gathered from standard X-rays with very little effort and can present a very valuable tool in the early detection of PHIT. 

Although the measurement of the parameters collected for this study is usually highly accurate when performed by experienced radiologists, the possibility of measuring errors does pose a limitation for this study. Repeated measurements or advanced radiographic imaging, such as additional MR images, might increase the accuracy of the results in future research on painful thumb joint instability. 

## 5. Conclusions

A high bony width offset of the os metacarpale I was identified as a significant risk factor for painful instability of the thumb basal joint in our study. The measurement of the slope angle did not show considerable differences between the study group and the control group. Both radiological signs are easily reproducible on standard radiographs in dp beam and zither player position. Radiographic imaging provides additional information and can be helpful in confirming the diagnosis of painful laxity in the CMC-1 joint. Furthermore, it can be used to identify patients that are at risk of developing thumb base laxity before they develop symptoms, so they can receive prophylactic treatment to prevent the condition from progressing into a debilitating and chronic state. It may also prevent the development of CMAOT.

This is, to our knowledge, the first study to specifically explore the slope angle and the bony offset as precursors for PHIT. Overall, the literature on PHIT is limited and very few studies report on these radiological findings in the context of risk classification or early detection.

The findings of our study underline the importance of thoroughly assessing hand radiographs. Special attention should be paid to the bony offset of the os metacarpale I, even if the patient does not complain of pain in the thumb joint. In doing so, asymptomatic individuals can be detected, and every affected patient can receive proper treatment. The aim is to spare them possibly avoidable symptoms and the need for excessive therapy in the future.

## Figures and Tables

**Table 1:** Baseline characteristics.

	Study Group	Control Group
**Gender**	30 female/3 male	13 female/22 male
**Age**	35.6 (s.e. 11.5)	34.7 (s.e. 10.7)
**Slope angle (mean)**	139.0 (s.e. 9.4)	139.9 (s.e. 9.6)
**Bony offset (mean)** in mm	2.03 (s.e. 2.31)	0.79 (s.e. 1.35)

**Table 2:** Probability for PHIT according to gender and bony offset.

Bony Offset (mm)	PHIT Prob (Female)	PHIT Prob (Male)
0	0.534	0.093
2	0.697	0.171
4	0.822	0.293
6	0.903	0.454

## Data Availability

Further information on the data presented in this study may be requested from the corresponding author. The original data sets are not publicly available due to privacy reasons.
